# Predictive value of TCR V**β**-J**β** profile for adjuvant gefitinib in *EGFR* mutant NSCLC from ADJUVANT-CTONG 1104 trial

**DOI:** 10.1172/jci.insight.152631

**Published:** 2022-01-11

**Authors:** Cunte Chen, Si-Yang Maggie Liu, Yedan Chen, Qiuxiang Ou, Hua Bao, Ling Xu, Yikai Zhang, Wenzhao Zhong, Qing Zhou, Xue-Ning Yang, Yang Shao, Yi-Long Wu, Si-Yang Liu, Yangqiu Li

**Affiliations:** 1 Key Laboratory for Regenerative Medicine of Ministry of Education, Institute of Hematology, School of Medicine, and; 2 Department of Hematology, First Affiliated Hospital, Clinical Medicine Postdoctoral Research Station, Jinan University, Guangzhou, China.; 3 Chinese Thoracic Oncology Group (CTONG), Guangzhou, China.; 4 Geneseeq Research Institute, Nanjing Geneseeq Technology Inc., Nanjing, China.; 5 Guangdong Lung Cancer Institute, Guangdong Provincial Key Laboratory of Translational Medicine in Lung Cancer, Guangdong Provincial People’s Hospital, Guangdong Academy of Medical Sciences, Guangzhou, China.; 6 School of Public Health, Nanjing Medical University, Nanjing, China.

**Keywords:** Oncology, Lung cancer, T cell receptor

## Abstract

Herein, we characterize the landscape and prognostic significance of the T cell receptor (TCR) repertoire of early-stage non–small cell lung cancer (NSCLC) for patients with an epidermal growth factor receptor (*EGFR*) mutation. β Chain TCR sequencing was used to characterize the TCR repertoires of paraffin-preserved pretreatment tumor and tumor-adjacent tissues from 57 and 44 patients with stage II/III NSCLC with an *EGFR* mutation treated with gefitinib or chemotherapy in the ADJUVANT-CTONG 1104 trial. The TCR diversity was significantly decreased in patients with an *EGFR* mutation, and patients with high TCR diversity had a favorable overall survival (OS). A total of 10 TCR Vβ-Jβ rearrangements were significantly associated with OS. Patients with a higher frequency of Vβ5-6Jβ2-1, Vβ20-1Jβ2-1, Vβ24-1Jβ2-1, and Vβ29-1Jβ2-7 had significantly longer OS. Weighted combinations of the 4 TCRs were significantly associated with OS and disease-free survival (DFS) of patients, which could further stratify the high and low TCR diversity groups. Importantly, Vβ5-6Jβ2-1, Vβ20-1Jβ2-1, and Vβ24-1Jβ2-1 had a significant relationship with gefitinib treatment, while Vβ29-1Jβ2-7 was associated with chemotherapy. Four TCR Vβ-Jβ rearrangements related to favorable OS and DFS for adjuvant gefitinib and chemotherapy in patients with an *EGFR* mutation with stage II/III NSCLC; this may provide a novel perspective for the adjuvant setting for resectable NSCLC.

## Introduction

Precision therapy for non–small cell lung cancer (NSCLC) based on genetic alterations greatly changed clinical practice (1, 2). Epidermal growth factor receptor (EGFR) tyrosine kinase inhibitors (TKIs) have become the standard of care for advanced NSCLC (3–6). The ADJUVANT-CTONG 1104 trial (Clinicaltrials.gov NCT01405079) demonstrated that the first-generation EGFR-TKI adjuvant gefitinib improves disease-free survival (DFS) from 19.8 months to 30.8 months for resected EGFR mutant NSCLC with N1/N2 metastasis; the subsequent ADAURA trial, the first international study with the third-generation EGFR-TKI osimertinib, reported positive outcomes for adjuvant TKIs in patients with *EGFR* mutations as well (7–10). The FDA approved osimertinib for adjuvant therapy in patients with *EGFR* exon 19 deletions or exon 21 *L858R* mutations (11).

Despite the success of targeted therapy in *EGFR* mutant NSCLC, the heterogeneous outcomes suggest that the effects may not depend solely on *EGFR*-driven cancer cells, but may also be associated with the local tumor immune microenvironment and antitumor T cell responses (12, 13). The latter may be characterized through the distribution and diversity of T cell receptors (TCRs) (14).

TCRs recognize tumor antigens derived by gene mutation or abnormal amplification and induce T cell activation through the clonal expansion of antigen-specific T cells, which may have antitumor activity (15–17). The 2 types of heterodimeric TCRs are α/β and γ/δ. Approximately 90%–95% of T cells in the blood are αβ^+^ T cells, and the rest are γδ^+^T cells. α/β TCRs recognize antigen peptides in the context of MHC molecules (18–20). Genes encoding the variable domains of TCR heterodimer chains are assembled by somatic rearrangement of one each of the variable (V), diversity (D, only for the β and δ chains), and joining (J) segments and comprise 3 hypervariable or complementarity-determining regions (CDR1, CDR2, and CDR3) in the TCR. Differences in the CDR3 in each TCR rearrangement result in a unique T cell clone that contributes to the diversity of TCR clones, which is known to be as high as 10^16–18^. In general, the distribution and diversity of β chain TCR (TCRβ) have been used to evaluate changes in the immune status of patients, and it could reflect the global picture of αβ^+^ T cells in healthy individuals and patients with cancer (21–23).

Previous studies have demonstrated that patients with cancer, including patients with NSCLC with higher numbers of clonally expanded T cells, have longer overall survival (OS) after checkpoint inhibitor immunotherapy (24–27). However, *EGFR* mutant NSCLC has an impaired response to immunotherapy. Evaluation of the diversity of the TCR repertoire either in blood T cells or tumor-infiltrating lymphocytes has been used to compare different responses to immune checkpoint inhibitors, such as PD-1 and PD-L1 blockades. Early TCR repertoire diversification after PD-L1 therapy for NSCLC may predict increased survival benefit (23, 24, 28). However, little is known about what influences the TCR repertoire in patients with NSCLC with an *EGFR* mutation and how the diversity and clonality of specific TCR rearrangements affect the efficacy of TKIs. If it can be defined, specific TCR profiles may be used as an immune biomarker for precise adjuvant TKI therapy and the prediction of clinical outcome or the selection of patients with EGFR mutation that could benefit from checkpoint blockade treatment.

In this study, based on the ADJUVANT-CTONG 1104 trial, we profiled the TCR repertoire of patients with an *EGFR* mutation with early-stage NSCLC and explored the predictive value of TCRβ clones for the different outcomes of the patients in the trial.

## Results

### High TCR clonality associated with favorable OS in patients with stage II/III NSCLC.

**Figure 1:**
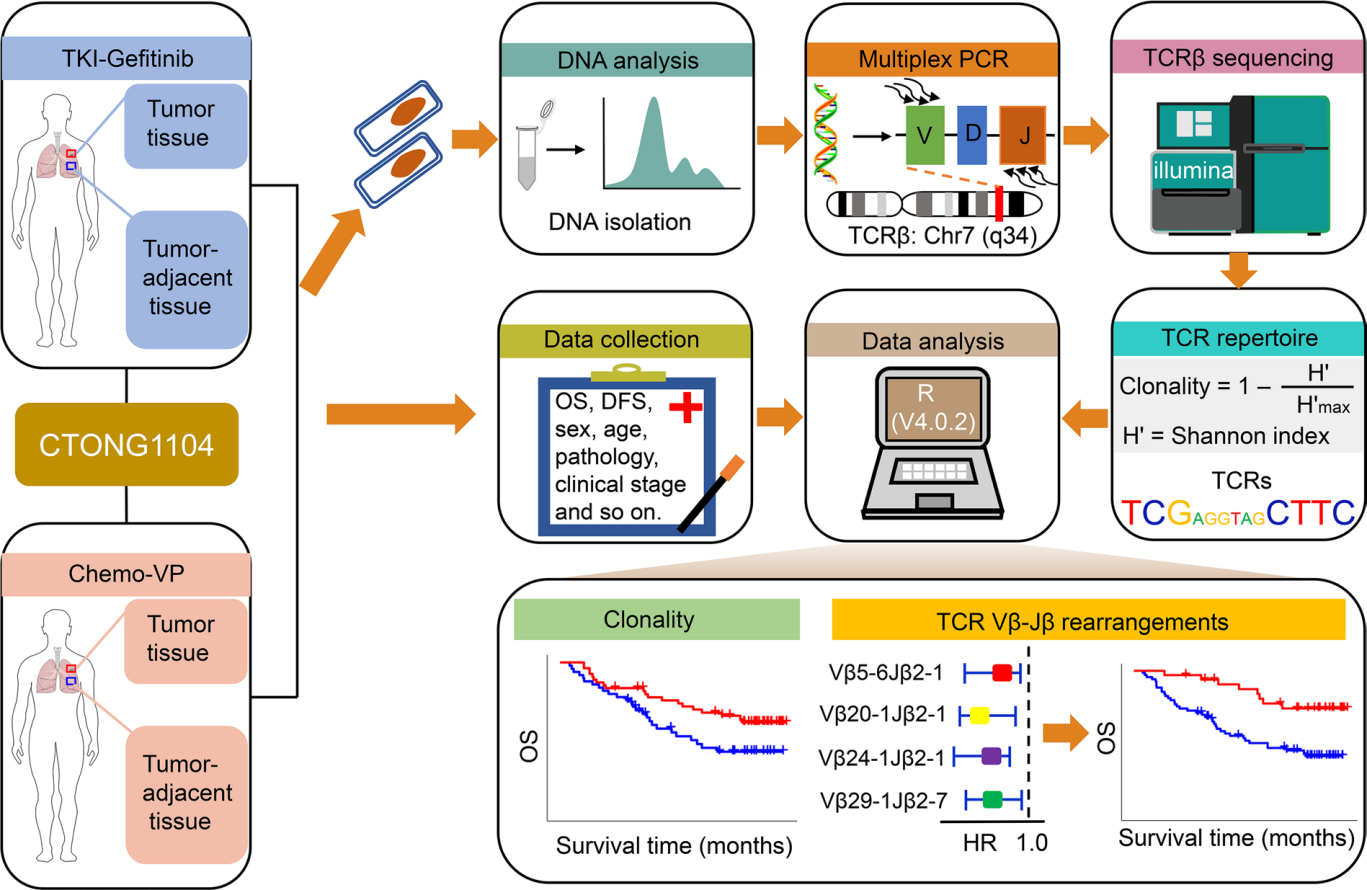
Study schematics. Patients from the ADJUVANT-CTONG 1104 trial with stage II/III NSCLC with an epidermal growth factor receptor (*EGFR*) mutation were divided into 2 cohorts to receive the *EGFR* tyrosine kinase inhibitor gefitinib (TKI-Gefitinib) or vinorelbine/cisplatin (Chemo-VP) treatment. The paraffin-preserved pretreatment tumor and tumor-adjacent tissues of patients in the TKI-gefitinib and Chemo-VP cohorts were collected and fractionated for DNA analysis and multiplex PCR for β chain T cell receptor (TCRβ) sequencing. Correlations between the TCR repertoire and clinical outcomes were explored.

A total of 222 patients from 27 sites across China were randomly divided 1:1 into 2 cohorts to receive gefitinib and vinorelbine plus cisplatin (VP) treatment, respectively (7–9). Pretreatment tumor tissues from 57 patients in the gefitinib cohort and 44 patients in the VP cohort were collected for TCRβ gene sequencing and prognostic analysis (Figure 1). Clinical characteristics were balanced between the 2 cohorts (Supplemental Table 1; supplemental material available online with this article; https://doi.org/10.1172/jci.insight.152631DS1).

**Figure 2:**
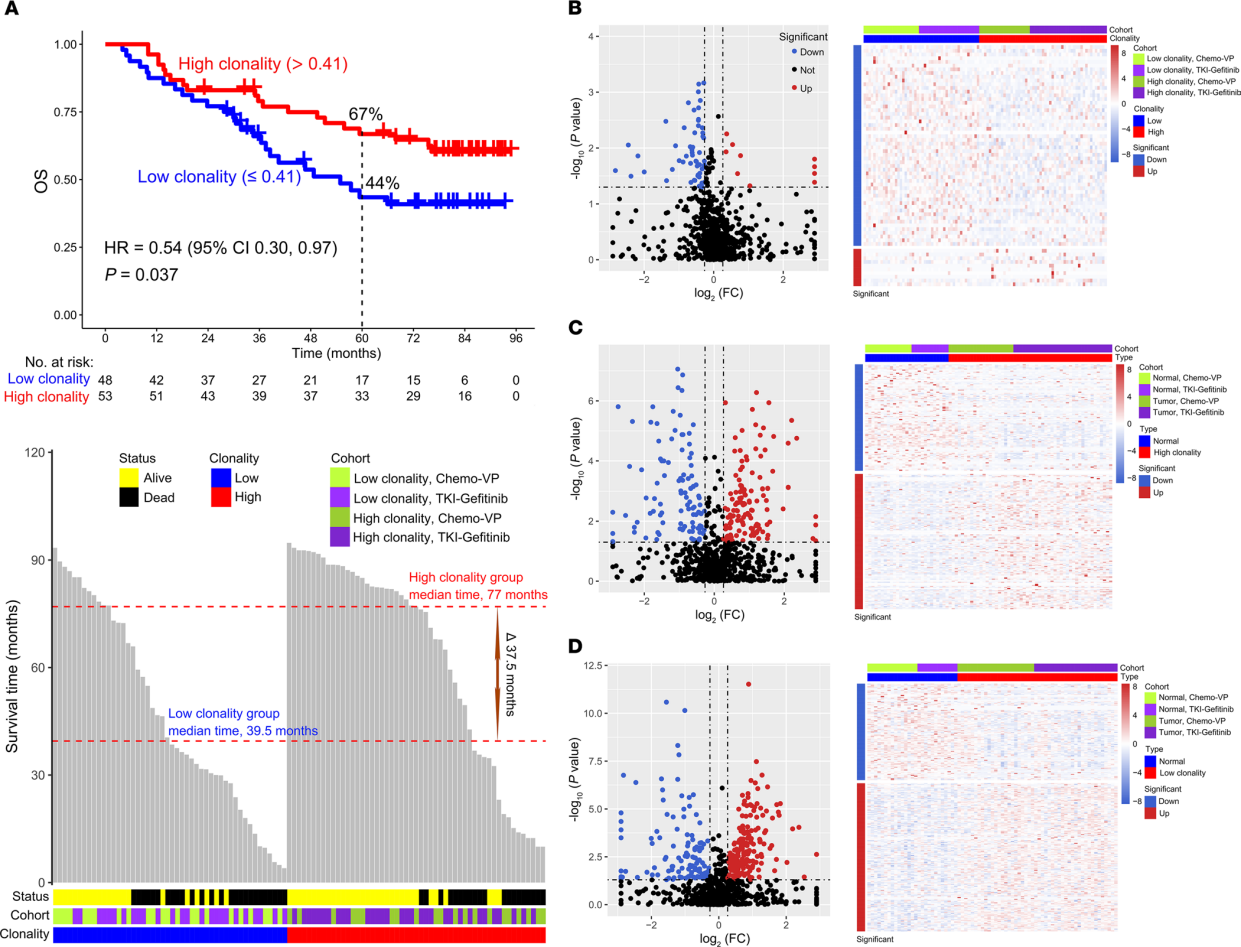
Overall survival analysis of patients with *EGFR* mutant lung cancer based on the clonality level. (**A**) Probability of overall survival (OS) for patients with high and low clonality (top). Distribution of the OS time of patients with low and high clonality (bottom). (**B**–**D**) Identification of differentially expressed TCRs by the Mann-Whitney-Wilcoxon test based on the high vs. low clonality groups (**B**), the high clonality group vs. tumor-adjacent tissue (**C**), and the low clonality group vs. tumor-adjacent tissue (**D**). Volcano plots show the differentially expressed TCRs between 2 groups (left). An absolute value of the fold change (FC) of more than 1.2 and a *P* value of less than 0.05 were used as a threshold to determine the significance of the differentially expressed TCRs. The red dots represent upregulated TCRs, the blue dots represent downregulated TCRs, and the black dots represent TCRs that did not change significantly. The heatmap shows the frequency of the differentially expressed TCRs based on an absolute value of fold change of more than 1.2 and a *P* value of less than 0.05 between 2 groups (right).

The TCR clonality was significantly lower in tumor tissues compared with normal-adjacent tumors (*P* < 0.001; Supplemental Figure 2). Considering that TCR clones reflect the T cell immune status, which may be related to the antitumor response and clinical outcome, we analyzed their relationship with OS. Based on the optimal cut-point of 0.41 (Supplemental Figure 1A), patients were divided into high and low TCR clonality groups. Patients with high TCR clonality had a better 5-year OS rate (*P* = 0.037; HR = 0.54; 95% CI, 0.30, 0.97; 5-year OS rate, high vs. low clonality, 67% vs. 44%) (Figure 2A, top) and longer median OS compared with patients with low TCR clonality (77 vs. 39.5 months) (Figure 2A, bottom).

To further identify TCRβ rearrangements related to patients with an *EGFR* mutation with stage II/III NSCLC, differential frequency of VJ usage was first analyzed. There were 10 frequently used and 54 less used rearrangements identified comparing high and low clonality groups (Figure 2B). Moreover, a total of 128 frequently used and 101 less used rearrangements were identified in the high TCR clonality group versus tumor-adjacent tissue, and 169 frequently used and 110 less used rearrangements were identified in the low TCR clonality group comparing to tumor-adjacent tissue (Figure 2, C and D). A total of 356 overlapping frequently and less used VJ rearrangements were used for subsequent analysis.

### The contribution of clonally expanded TCRs to prognostic attributes for stage II/III NSCLC.

**Figure 3:**
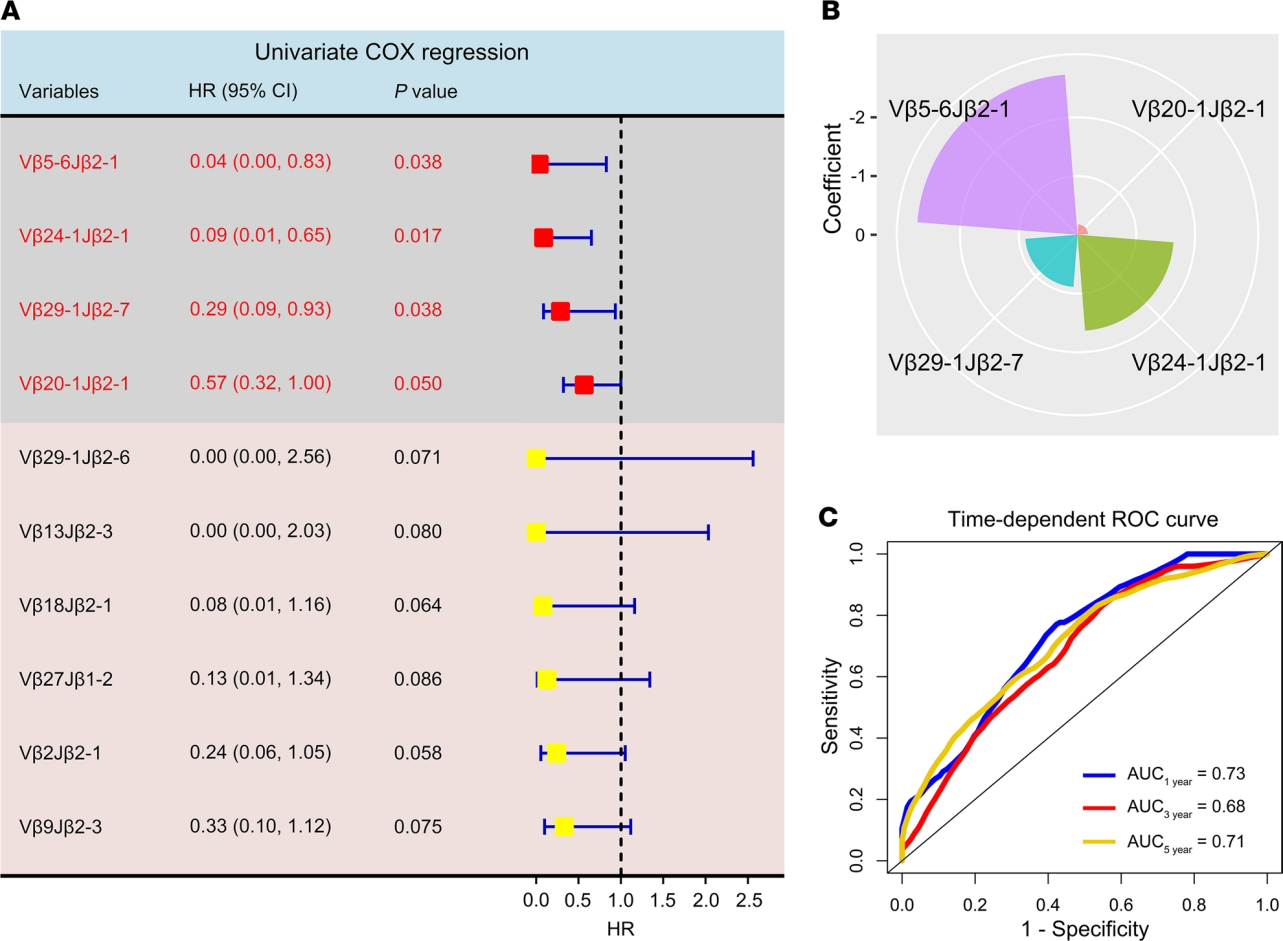
Uni- and multivariate COX regression analysis of differentially expressed TCRs. (**A**) Forest plot of univariate COX regression analysis showing that clonal TCRs are associated with favorable overall survival (OS) based on *P* < 0.1. The statistically significant TCRs are shown in red, with a *P* ≤ 0.050. (**B**) The radar plot shows the contribution of the 4 TCRs to OS, which was determined by the coefficients of the 4 TCRs in the multivariate COX regression model. The smaller areas represent a lower contribution, whereas the larger areas represent a greater contribution. (**C**) A time-dependent receiver operating characteristic (ROC) curve plotted by the “survivalROC” package in R (version 4.0.2) was used to evaluate the performance of the multivariate COX regression model. AUC of more than 0.5 indicated that the model had better performance.

Considering the importance of T cells in the antitumor response in patients with an *EGFR* mutation with early-stage NSCLC, we explored their relationship with clinical outcomes. Remarkably, we identified 10 TCR Vβ-Jβ rearrangements associated with favorable OS by univariate COX regression analysis of VJ frequencies (*P* < 0.1). Four of these rearrangements demonstrated the predefined statistical significance (*P* ≤ 0.050, Figure 3A). Multivariate COX regression of these 4 TCR Vβ-Jβ rearrangements suggested that TCR Vβ5-6Jβ2-1 had the highest contribution to OS (coefficient = –2.733). Importantly, according to the coefficients obtained from the multivariate COX regression model, the following formula can be used to calculate the risk score of each patient: risk score = –2.733 × (frequency of Vβ5-6Jβ2-1) –0.186 × (frequency of Vβ20-1Jβ2-1) –1.638 × (frequency of Vβ24-1Jβ2-1) –0.891 × (frequency of Vβ29-1Jβ2-7) (Figure 3B). The time-dependent receiver operating characteristic curve (ROC) was used to further evaluate the performance of the multivariate COX regression model, and the results indicated that the estimated risk score could well reflect the prognosis of patients (AUC, 1 yr vs. 3 yr vs. 5 yr, 0.73 vs. 0.68 vs. 0.71, respectively) (Figure 3C). Taken together, a weighted combination of these special TCR Vβ-Jβ rearrangements had the potential for risk stratification of patients with *EGFR* mutant NSCLC.

### Weighted combinations of TCRβs associated with clinical outcome.

**Figure 4:**
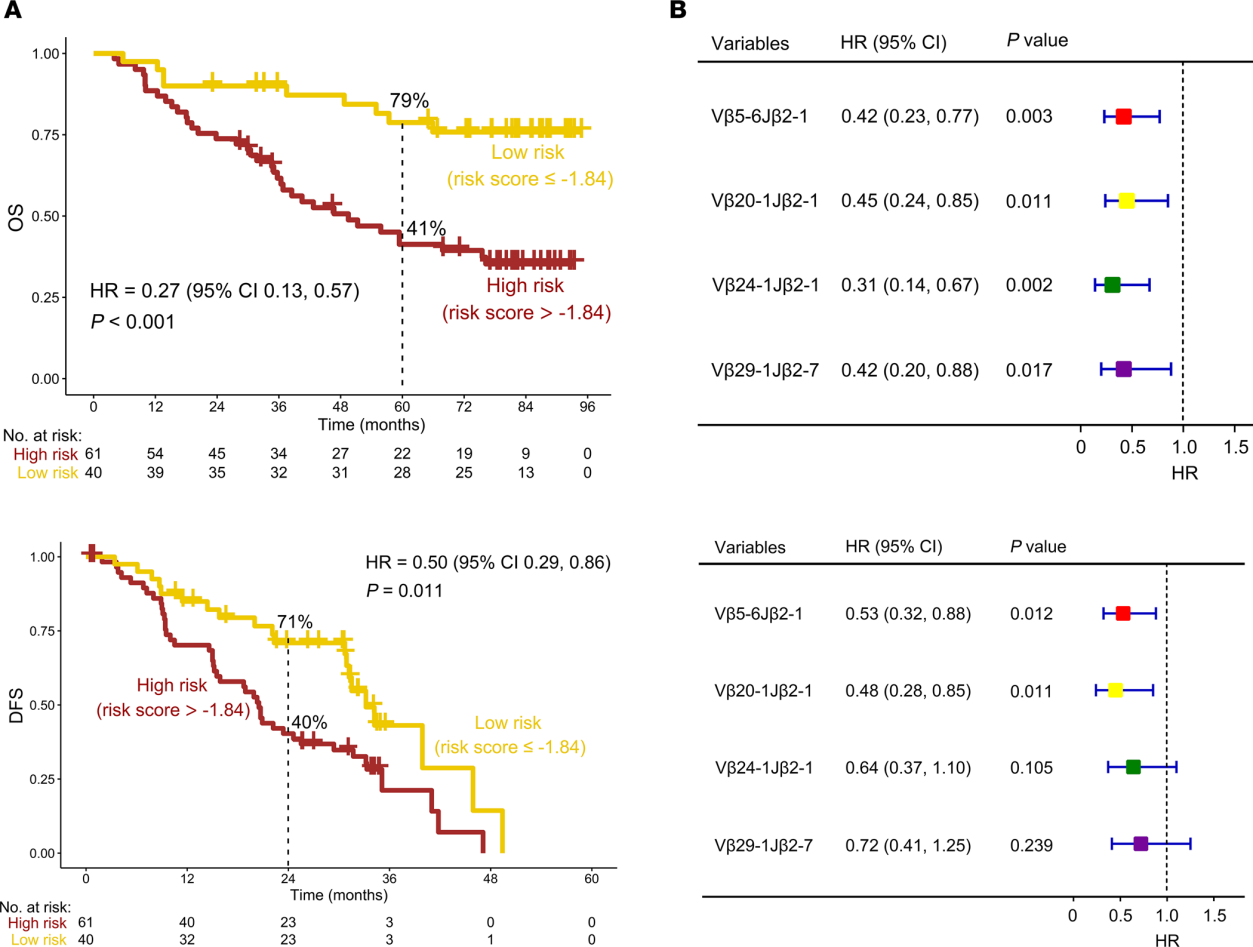
A weighted combination of 4 TCRs was associated with the prognosis of patients with an *EGFR* mutation with NSCLC. (**A**) Overall survival (OS) (top) and disease-free survival (DFS) (bottom) analysis of low- and high-risk patients based on the combination of 4 TCRs. (**B**) OS (top) and DFS (bottom) analysis of patients with a low and high frequency of Vβ5-6Jβ2-1, Vβ20-1Jβ2-1, Vβ24-1Jβ2-1, and Vβ29-1Jβ2-7 by Kaplan-Meier analysis.

We next sought to assess the relationship between the weighted combinations of Vβ5-6Jβ2-1, Vβ20-1Jβ2-1, Vβ24-1Jβ2-1, and Vβ29-1Jβ2-7 and survival outcomes. Patients with stage II/III NSCLC with an *EGFR* mutation were divided into low- and high-risk groups according to the optimal cut-point for the risk score (cut-point = –1.84; Supplemental Figure 1F). Interestingly, low-risk patients had significantly longer OS compared with high-risk patients (*P* < 0.001; HR = 0.27; 95%CI, 0.13, 0.57; 5-year OS rate, low- vs. high-risk, 79% vs. 41%) (Figure 4A, top). Of note, favorable OS with a low-risk score was observed in both high- and low-clonality subgroups (*P* < 0.01; Supplemental Figure 3). Importantly, patients with low risk also showed longer DFS than those with high risk (*P* = 0.011; HR = 0.50; 95%CI, 0.29, 0.86; 2-year DFS rate, low vs. high risk, 71% vs. 40%) (Figure 4A, bottom). In order to determine whether the weighted combination of these 4 TCR clones can predict the prognosis of stage II/III patients with an *EGFR* mutation with NSCLC, we performed univariate and multivariate Cox proportional hazard regression analyses. Interestingly, when sex, age, smoking history, pathology, clinical stage, N stage, treatment options, and risk score were included in the multivariate COX regression model for survival analysis, the results suggested that risk score was an independent prognostic predictor for OS (*P* < 0.001; HR = 0.25; 95%CI, 0.12, 0.54). This finding was confirmed in DFS analysis (*P* = 0.026; HR = 0.53; 95%CI, 0.30, 0.93) (Table 1).

Because of the prognostic importance of TCR rearrangements, their relationship with OS was further investigated. Patients were divided into high- and low-frequency groups based on the optimal cut-points for the TCRs (Supplemental Figure 1, B–E). The results of Kaplan-Meier survival analysis demonstrated that a high frequency of Vβ5-6Jβ2-1 and Vβ20-1Jβ2-1 was significantly associated with favorable OS and DFS (*P* < 0.05). However, the high frequency of Vβ24-1Jβ2-1 and Vβ29-1Jβ2-7 only correlated with favorable OS but not with DFS (*P* > 0.1, Figure 4B). These results provide support for the hypothesis that the special TCR rearrangement status of infiltrated T cells in tumors may influence the efficacy of treatment.

**Figure 5:**
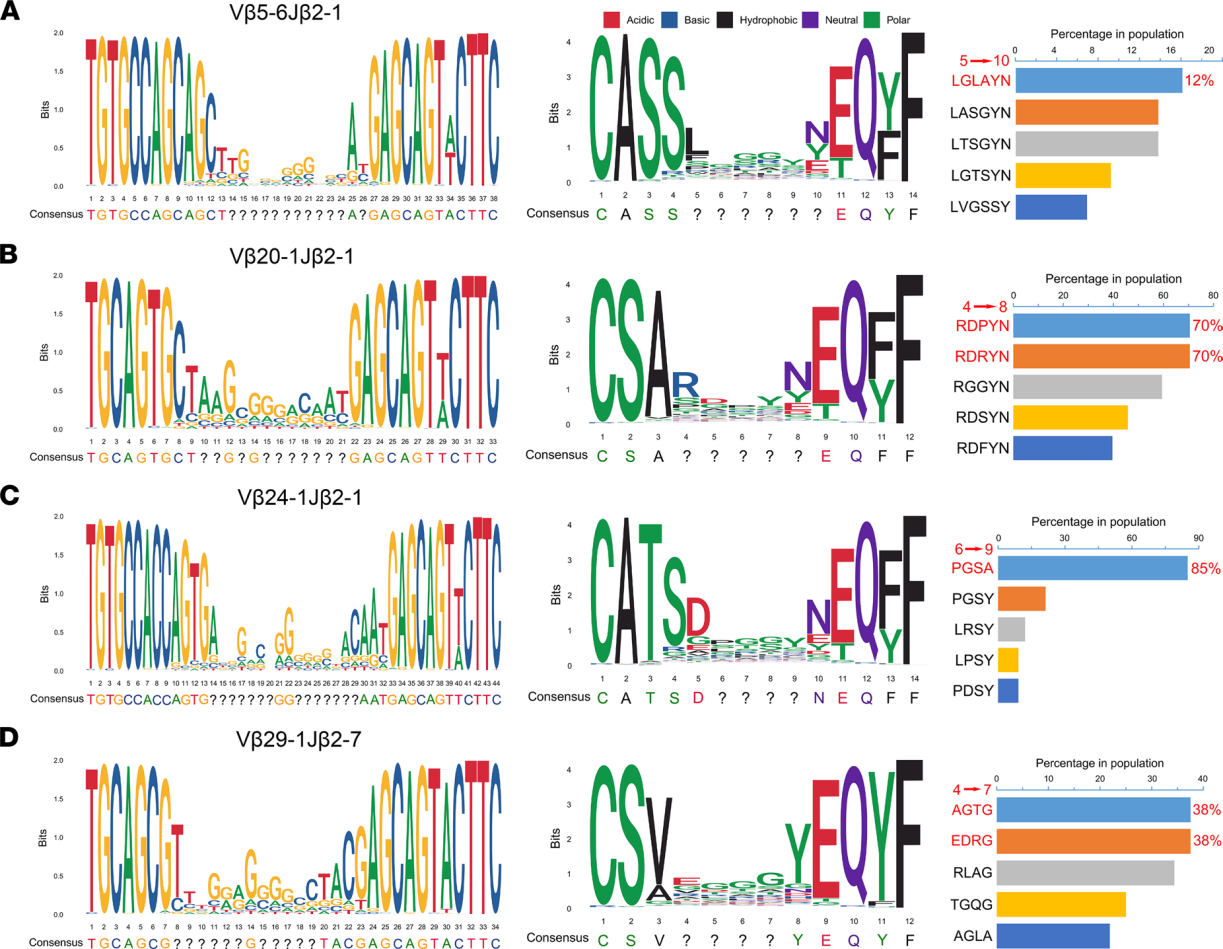
Sequence motif analysis of high-frequency groups of TCRs. Local alignment was used to calculate the similarity of the base (left) and amino acid (middle) sequences of Vβ5-6Jβ2-1 (**A**), Vβ20-1Jβ2-1 (**B**), Vβ24-1Jβ2-1 (**C**), and Vβ29-1Jβ2-7 (**D**), which were identified by the “msa” package and plotted by the “ggplot2” and “ggseqlogo” packages in R. The question marks indicate that the sequence at that position is not conservative. The proportion of the top 5 nonconservative amino acid sequences in patients with a high frequency of TCRs is also shown (right).

To identify clonotype contributions of these 4 TCR rearrangements, we further investigated the nucleotide and amino acid sequence motifs of the 4 TCRs in the high-frequency TCR groups. The results revealed that the nucleotides and amino acids at both ends of the CDR3 region were almost completely conserved and probably belong to the Vβ and Jβ segments joining the N nucleotides and the Dβ segments of TCR (Figure 5, left and middle columns). However, the sequences and lengths of the CDR3 regions appeared to be diverse. It is known that the CDR3 of TCRs is the region responsible for antigen recognition by T cells, and it represents the specific response of a T cell, such as an antitumor effect. However, the difference of CDR3 sequences from the same TCR Vβ-Jβ rearrangement may be associated with the restriction of HLA subtypes in different patients. Therefore, we sought to collect the top 5 frequent CDR3 sequences of these 4 rearrangements in the population of high-frequency groups that potentially represented common CDR3 motifs responding to tumor-associated NSCLC antigens in this study. The amino acid sequence of Vβ5-6Jβ2-1 with the highest proportion in the population was LGLAYN, which accounted for only 12%. Intriguingly, the sequences RDPYN and RDRYN in Vβ20-1Jβ2-1 accounted for a relatively high population proportion, reaching 70%. Moreover, up to 85% of patients with a high-frequency Vβ24-1Jβ2-1 had the sequence PGSA. However, only 38% of patients with a high-frequency Vβ29-1Jβ2-7 had the sequences AGTG and EDRG (Figure 5, right column).

### Identification of adjuvant treatment-related indicator TCRs.

**Figure 6:**
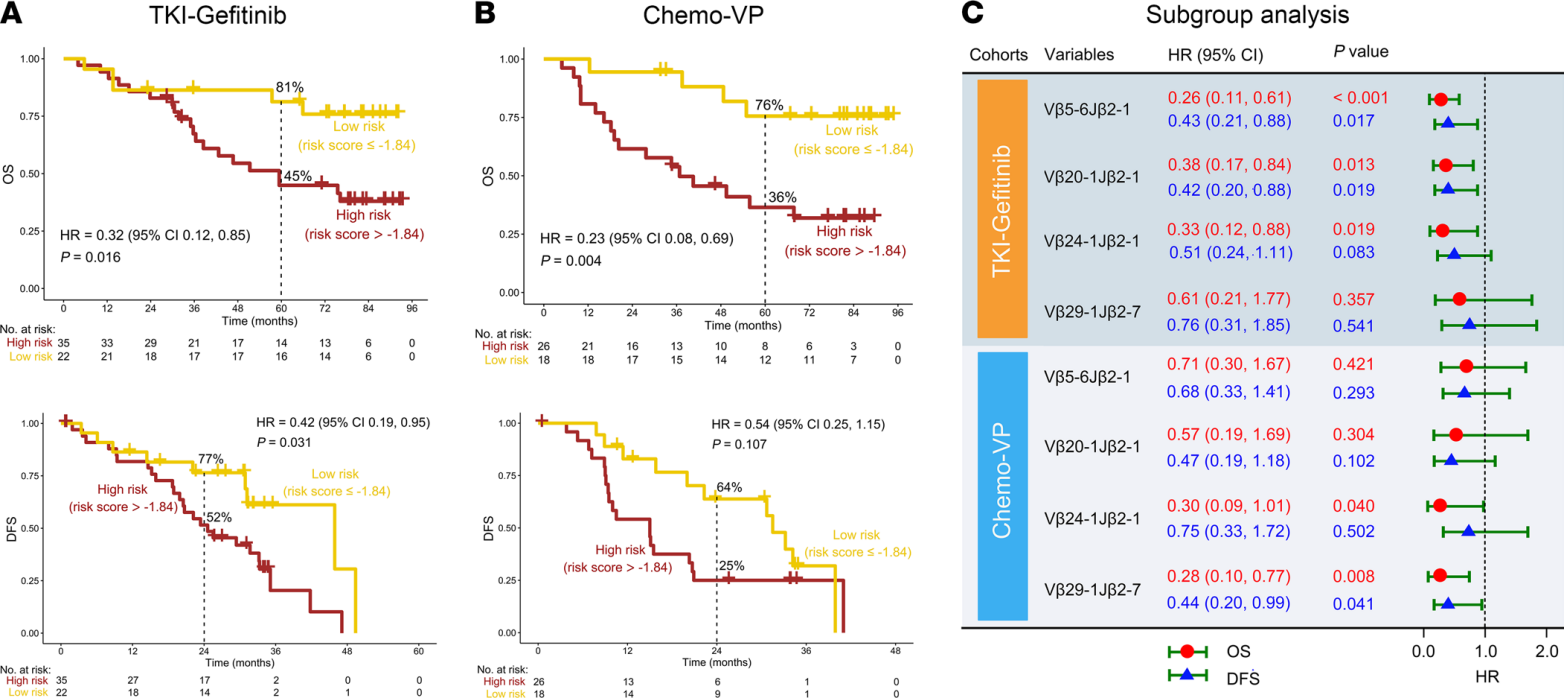
Kaplan-Meier survival analysis of TCRs in the TKI-gefitinib and Chemo-VP cohorts. A weighted combination of 4 TCRs for overall survival (OS) (top) and disease-free survival (DFS) (bottom) analysis in the TKI-gefitinib (**A**) and Chemo-VP (**B**) cohorts. (**C**) OS and DFS analysis of Vβ5-6Jβ2-1, Vβ20-1Jβ2-1, Vβ24-1Jβ2-1, and Vβ29-1Jβ2-7 by Kaplan-Meier analysis in the TKI-gefitinib and Chemo-VP cohorts.

According to the above findings, Vβ5-6Jβ2-1 and Vβ20-1Jβ2-1 were both significantly associated with favorable OS and DFS, and Vβ24-1Jβ2-1 and Vβ29-1Jβ2-7 only correlated with favorable OS but not with DFS in patients with stage II/III NSCLC with an *EGFR* mutation regardless of adjuvant gefitinib or chemotherapy. We were then interested in further characterizing whether these TCR rearrangements could inform treatment selection for adjuvant TKI or chemotherapy. The results demonstrated that low-risk patients had a longer OS than high-risk patients in the *EGFR* TKI gefitinib (TKI-gefitinib) cohort (*P* = 0.016; HR = 0.32; 95% CI, 0.12, 0.85; 5-year OS rate; low vs. high risk, 81% vs. 45%) (Figure 6A, top). Remarkably, when analyzing the relationship between risk score and DFS, similar findings could be observed (*P* = 0.031; HR = 0.42; 95% CI, 0.19, 0.95; 2-year DFS rate, low vs. high risk: 77% vs. 52%) (Figure 6A, bottom). Interestingly, high frequency of Vβ5-6Jβ2-1 and Vβ20-1Jβ2-1 was significantly correlated with both favorable OS and DFS (*P* < 0.05), while an association between Vβ29-1Jβ2-7 and OS and DFS in the TKI-gefitinib cohort was not found (*P* > 0.05). In addition, patients with a high frequency of Vβ24-1Jβ2-1 had a favorable OS (*P* = 0.019) but no significant relationship with DFS (*P* = 0.083) in the TKI-gefitinib cohort (Figure 6C). However, in the vinorelbine/cisplatin (Chemo-VP) cohort, low-risk patients were associated with favorable OS (*P* = 0.004; HR = 0.23; 95% CI, 0.08, 0.69; 5-year DFS rate, low vs. high risk: 76% vs. 36%) but not DFS (*P* = 0.107) (Figure 6B). Surprisingly, when we analyzed the relationship between TCRs and OS and DFS in the Chemo-VP cohort, the results were the opposite of those found in the TKI-gefitinib cohort. Vβ5-6Jβ2-1 and Vβ20-1Jβ2-1had no significant relationship with OS and DFS (*P* > 0.05), while a high frequency of Vβ29-1Jβ2-7 was associated with both favorable OS and DFS (*P* < 0.05). In addition, patients with high frequency of Vβ24-1Jβ2-1 had a favorable OS (*P* = 0.040) but had no significant relationship with DFS (*P* = 0.502) (Figure 6C). Overall, clonally expanded Vβ5-6Jβ2-1, Vβ20-1Jβ2-1, and Vβ24-1Jβ2-1 might be beneficial for resectable early-stage patients with NSCLC choosing TKI therapy as adjuvant treatment, while clonally expanded Vβ29-1Jβ2-7 may be an indicator for patients with an *EGFR* mutation choosing chemotherapy as adjuvant treatment.

## Discussion

The results of the ADJUVANT-CTONG 1104 trial showed that, compared with chemotherapy and historical data, patients with stage II/III NSCLC with an *EGFR* mutation who receive adjuvant therapy with gefitinib had improved DFS and the longest OS (8, 29). However, various factors may contribute to the differences in treatment effects. Among them, the immune status of patients is a factor that cannot be ignored, particularly T cell clonal expansion and TCR repertoire diversity. There is increasing evidence that T cell clone expansion and high TCR diversity are significantly associated with the response of immune checkpoint inhibitors and favorable prognosis in a variety of patients with cancer (25–27, 30). Thus, we wondered how immune status influenced the outcome of patients in the ADJUVANT-CTONG 1104 trial (8, 29).

Based on high-throughput TCRβ sequencing analysis of samples from the ADJUVANT-CTONG 1104 trial, we found that the diversity of TCR rearrangements was significantly decreased in patients with stage II/III NSCLC with an *EGFR* mutation, and patients with high TCR diversity had favorable OS. These findings are consistent with those of previous studies that reported changes in TCR diversity in patients with advanced lung cancer (26, 31). However, importantly, systematic analysis of the TCR repertoire landscape and the characteristics of the relationship between TCR diversity and OS as well as DFS are lacking. In particular, the global distribution of TCR diversity and identification of outcome-related TCR clones have not been described in NSCLC based on clinical trial data. In this study, we characterized the landscape of the TCRβ repertoire in tumor tissue from patients with stage II/III NSCLC with an *EGFR* mutation who received adjuvant therapy, including the TKI gefitinib and VP in the clinical trial (8). In general, random recombination of the TCRα and β V(D)J regions will generate a diverse TCR repertoire, with as many as 10^16–18^ TCRs that possess specific recognition capability for antigen peptides presented by antigen-presenting cells (32, 33). However, the presence of dominant tumor clones and decreased TCR diversity were commonly observed in patients with cancer (26, 34, 35). As expected, in this study, we also found that the diversity of the TCR repertoire in tumor tissues was significantly decreased. Indeed, lower TCR diversity is related to the reduced potential ability of T cell activity to respond to different antigens. In contrast, the reason for the lower TCR diversity may be due to the increase in clonally expanded TCRs. The latter could be detected in patient peripheral blood and tumor-infiltrating lymphocytes, possibly as T cell clones responding to tumor-associated antigens and tumor-specific antigens, and it may play a critical role in antitumor activity (32, 36, 37). In this study, we showed that a total of 10 clonal TCRs are associated with OS; confirmed the significance of 4 of the 10 TCR clones, including Vβ5-6Jβ2-1, Vβ20-1Jβ2-1, Vβ24-1Jβ2-1, and Vβ29-1Jβ2-7; and characterized the different contributions of TCRs to OS and DFS. Moreover, according the results of univariate and multivariate Cox proportional hazard regression analyses, we can confirm that weighted combination of these 4 TCR clones is an independent prognostic predictor for stage II/III patients with an *EGFR* mutation with NSCLC. To our best knowledge, there have been no reports demonstrating an association between special TCR rearrangements and the OS and DFS of patients with NSCLC. These findings might provide new insight for prospective trials using specific TCRs as biomarkers, which will further validate the function of identified TCR clones. Currently, we are unable to demonstrate the antitumor activity of these 4 TCRs. However, we also tried to find evidence to support our finding. Interestingly, Echchakir et al. have reported high frequencies of Vβ5, Vβ20, and Vβ24 in patients with NSCLC (38). A previous work of ours demonstrated predominant expanded Vβ20-1 and Vβ29-1 in healthy human CD3^+^ T cells after WT1-specific antigen peptide or antigen BCR-ABL peptides induction (39). Notably, Vβ5 clone CD8^+^ T cells were related to antitumor activity in melanoma (40). Therefore, with the findings that these TCR clones were associated with favorable clinical outcomes in patients with an *EGFR* mutation with NSCLC, we suggest that the identified 4 TCR clones might have an antitumor response in NSCLC. We will conduct experiments in the future to validate the relationship between these 4 specific TCR rearrangements and the antitumor response in *EGFR* mutant NSCLC.

Our previous studies have shown that patients with an *EGFR* mutation who receive adjuvant treatment, including gefitinib, have better DFS than those receiving chemotherapy (8, 29). Therefore, we attempted to find a predictive value for the TCRs identified in the TKI-gefitinib and Chemo-VP subgroups. Interestingly, we found that Vβ5-6Jβ2-1, Vβ20-1Jβ2-1, and Vβ24-1Jβ2-1 had a significant relationship with gefitinib treatment, while Vβ29-1Jβ2-7 was associated with chemotherapy. We suggest that these 4 TCRs might be predictive biomarkers for the treatment effects of patients with stage II/III NSCLC with an *EGFR* mutation who received adjuvant treatment with gefitinib or chemotherapy.

It is known that tumor-associated TCR clones are not only used for evaluating immune status but also for the development of special immunotherapeutic treatments based on TCR-redirected T cells (TCR–T cells) (41–43). In this regard, the sequences of such expanded TCR clones need to be identified. In this study, we also identified frequent CDR3 sequences in the 4 TCRs that are essential for the specificity and affinity of antigen recognition with HLA restriction. There are reports showing specific TCRβ sequences derived from patients with NSCLC, including HLA-A*02:01/CT37 peptide-specific α and β TCR chains, in a CD8^+^ T cell clone (44). However, these findings are only from few cases. In this study, we identified TCR clones based on a large cohort trial, which may illustrate the common features of the clinically expanded TCR repertoire and could be used as biomarkers for clinical outcome evaluation. Overall, we identified TCR CDR3 sequences that might be tumor antigen-specific TCRs in patients, which may be further used as biomarkers and could be isolated by cloning and constructing TCR–T cells for immunotherapy (43, 45–47).

In summary, in this exploratory analysis of the ADJUVANT-CTONG 1104 trial, high TCR diversity was significantly associated with favorable OS for patients with stage II/III NSCLC with an *EGFR* mutation. To our best knowledge, we, for the first time, identified that 4 special TCR Vβ-Jβ rearrangements, Vβ5-6Jβ2-1, Vβ20-1Jβ2-1, Vβ24-1Jβ2-1, and Vβ29-1Jβ2-7, are related to TKI-gefitinib and Chemo-VP treatment. These findings may provide a new perspective for prospective clinical trials and the immunotherapy of patients with stage II/III NSCLC with an *EGFR* mutation. The limitations of this study include the lack of an external validation cohort; there is no public or clinical data set equivalent to that use in this study with regular long-term follow-up and TCRβ sequencing in patients with stage II/III NSCLC with an *EGFR* mutation who received adjuvant therapy, including gefitinib and chemotherapy. Moreover, single-cell RNA sequencing and TCR sequences can trace the phenotype of such expanded TCR clones as well as their function in transcriptomes level. We will try to perform single-cell RNA and TCR sequencing to define the TCR clones in selected samples in the future. Furthermore, further investigation will be performed to analyze the T cell subtypes to which these special TCR Vβ-Jβ clones belong and validate their anti-NSCLC effects by sorting the T cell subtypes that express the clonal TCRs or constructing engineered TCR–T cells in the future.

## Methods

### Patients.

In the ADJUVANT-CTONG 1104 trial, patients between the ages of 18 and 75 years with completely resected (R0), stage II/IIIA (N1–N2), *EGFR* mutant (exon 19 deletion or exon 21 Leu858Arg) NSCLCs were included and randomized for adjuvant gefitinib or VP treatment. Gefitinib was given at 250 mg once daily for 24 months, and intravenous vinorelbine and cisplatin were administered at 25 mg/m² and 75 mg/m² on day 1 every 3 weeks for 4 cycles (7–9). We prospectively collected resected tumors and tumor-adjacent tissues from 57 patients treated with gefitinib and 44 patients treated with VP chemotherapy.

### TCR library preparation and sequencing.

Archived FFPE blocks of tumor tissues were obtained from the ADJUVANT trial. Five to eight 10 μm FFPE sections were first deparaffinized with xylene and then used for genomic DNA (gDNA) extraction with the QIAamp DNA FFPE Tissue Kit (Qiagen), according to the manufacturer’s instructions. The extracted gDNA samples were quantified with a Qubit 3.0 fluorometer (Thermo Fisher Scientific), and their purity was measured with a Nanodrop 2000 (Thermo Fisher Scientific). A minimum of 1 μg DNA was required for subsequent experiments. Only patients with sufficient and qualified tumor tissue for TCR sequencing were included in this study, resulting in 57 tumor tissues and 12 tumor-adjacent tissues from the gefitinib arm, and 44 tumor tissues and 15 tumor-adjacent tissues from the chemotherapy arm. A multiplex PCR reaction was prepared using the Qiagen Multiplex PCR Plus Kit with a customized TCR primer mixture comprising 51 forward primers complementary to the V gene segments and 13 reverse primers complementary to the J gene segment. To correct for amplification bias from the multiplex PCR primers, 663 barcoded synthetic templates (i.e., a synthetic repertoire of all possible V-J combinations) were used to calibrate the PCR efficiency. These templates contain universal P5 and P7 ends for standard primer recognition, barcodes, and V and J gene segments flanking barcoded internal markers. Amplified synthetic products and tumor samples were then purified using the AxyPrep MAG FragmentSelect-I Kit (Axygen). Subsequently, the tumor TCR library was prepared with the KAPA Hyper Prep Kit (KAPA Biosystems). Briefly, A-tailing and end-repair of fragments were performed before the ligation of index adaptors from the TruSeq DNA PCR-free Library Prep Kit (Illumina). Purified ligation products were then amplified with Illumina p5 and p7 primers with KAPA HiFi HotStartReadyMix (KAPA Biosystems), followed by a final purification step using Axygen beads. TCR libraries were sequenced using the Illumina HiSeq 4000 platform according to the manufacturer’s instructions.

### TCR analysis and profiling.

Trimmomatic was used to remove adaptors and filter low-quality reads from FASTQ files. Non–V-J paired reads were further removed using Cutadapt (V 1.18). Next, paired-end read merger (PEAR, V 0.9.10) was employed to merge paired reads, and nonbarcoded reads were removed for synthetic standards, while barcoded reads were filtered for tumor samples. Clean reads were subsequently assembled using MiXCR (V 2.1.11). Reads were aligned to reference V or J gene segments according to the international ImMunoGeneTics database. Clonotypes, defined as unique antigen-recognizing CDR3 sequences assembled from specific usage of VJ gene segments, were then built from the alignments using the assemble pipeline of the software. TCR clonality describes the diversity of clonotypes of a TCR population. For final repertoire profiling, sample V/J counts, CDR3 counts, and clonality counts were calculated with normalization using corresponding counts from synthetic standards.

TCR clonality analysis was performed using common diversity measures from the R package vegan (48). Specifically, the diversity of the TCR repertoire was assessed by the Shannon index (*H*):

H = – Σpilnpi,

where *pi* is the proportion of individuals found in the *i*th species and *ln* represents the natural logarithm (49). Clonality was defined as 1-Pielou’s Evenness (*J*), where *J* is normalized Shannon’s entropy to measure the distribution of the TCR population calculated by dividing the Shannon index (*H’*) by the total number of species in a sample (*S*):


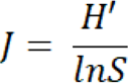


### Selection of TCR rearrangements.

To explore the association of specific TCR rearrangements with treatment outcomes, patients were first categorized into high and low diversity groups according to the optimal prognostic cutoff of TCR clonality that most significantly separated patient outcomes. Differential usage of VJ rearrangements was then compared between high and low diversity tumors and between each diversity subgroup and tumor-adjacent tissues, using the frequency count of each unique VJ rearrangement. Only rearrangements with absolute fold change of more than 1.2 and a Mann-Whitney-Wilcoxon test *P* value of less than 0.05 were selected for further analysis.

### Statistics.

The optimal prognostic cut-points for TCR diversity, TCR rearrangements, and risk scores were determined by maximally selected rank statistics in the “maxstat” package. This is an outcome-oriented method that identifies a cut-point that best separates survival outcomes (50–53) (Supplemental Figure 1). Differential VJ rearrangements were identified using the “limma” package (54). Kaplan-Meier curves of DFS and OS were compared by the log-rank test (55). Comparison of the 2 groups of quantitative variables was conducted by the Mann-Whitney-Wilcoxon test. Univariate and multivariate Cox proportional hazard regression analyses were conducted by the “survival” package (56). Differences between qualitative variables were compared by the χ^2^ and Fisher’s exact tests as appropriate. A 2-tailed *P* < 0.05 was considered statistically significant. The consensus of local nucleotide and amino acid sequences in the CDR3 region was analyzed by aligning and calculating sequence similarity with the “msa” package (57) and plotted with the “ggseqlogo” packages (58). All statistical analyses and graphical illustrations were performed with R software (version 4.0.2, https://www.r-project.org/).

### Study approval.

This study was conducted according to the principles of the Declaration of Helsinki and was approved by the ethics committee of Guangdong Provincial People’s Hospital (no. 2011713). Written informed consent was received from participants prior to inclusion in the study. Participants were identified by number, not by name.

## Author contributions

YL, YLW, and SYL contributed to the concept development and study design and edited the manuscript. CC and SYML interpreted the data and wrote the manuscript. YC drafted the Methods and revised the manuscript. QO developed the TCR test and initial data analysis. HB provided initial bioinformatic data analysis and reviewed the statistical methodologies and results. LX and YZ interpreted the data. WZ, QZ, and XNY diagnosed and treated the patients and provided clinical samples. YS supervised data analysis and manuscript editing. All authors read and approved the final manuscript.

## Supplementary Material

Supplemental data

## Figures and Tables

**Table 1:**
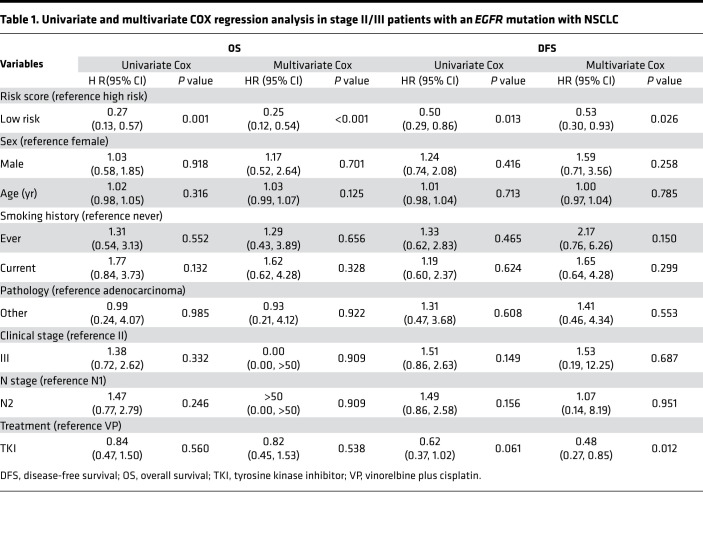
Univariate and multivariate COX regression analysis in stage II/III patients with an *EGFR* mutation with NSCLC

## Version 1 01/11/2022

Electronic publication
