# Supplementary material for: Predictive value of TCR Vβ-Jβ profile for adjuvant gefitinib in EGFR mutant NSCLC from ADJUVANT-CTONG 1104 trial
Source: JCI Insight. 2022 Jan 11;7(1):e152631. doi: 10.1172/jci.insight.152631 (PMC8765044; doi:10.1172/jci.insight.152631)
Supplement: Supplemental data [file jciinsight-7-152631-s109.pdf]

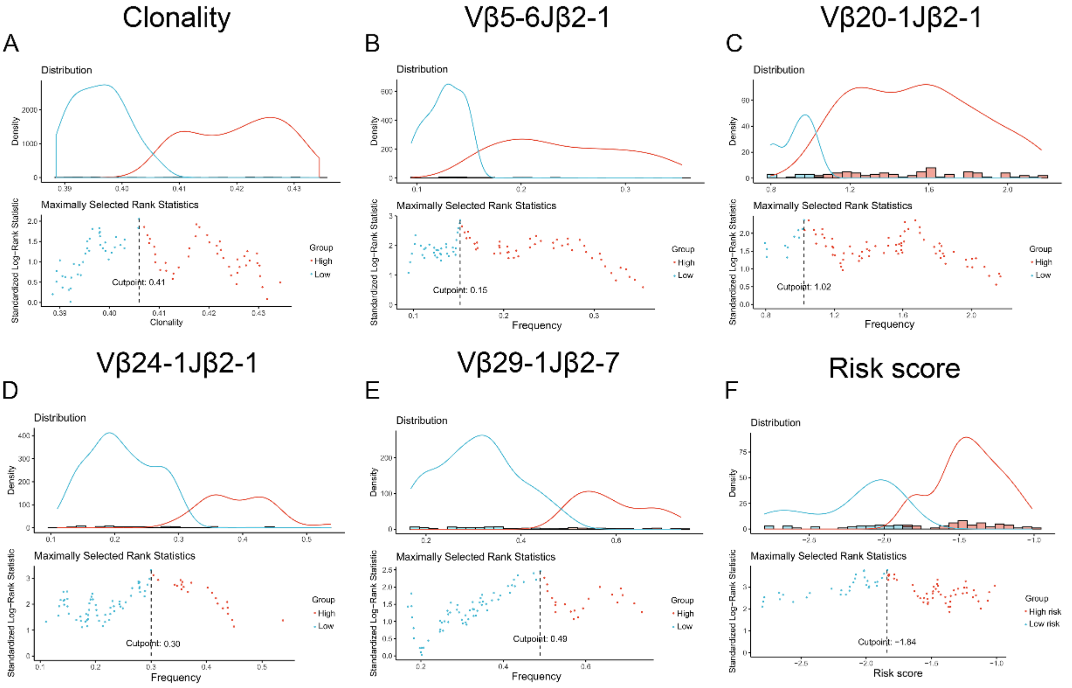

Figure 1. The maximally selected rank statistics from the “maxstat” package in R (version 4.0.2, https://www.r-project.org/) was used to determine the optimal cut-points for clonality **(A)**, Vβ5-6Jβ2-1 **(B)**, Vβ20-1Jβ2-1 **(C)**, Vβ24-1Jβ2-1 **(D)**, Vβ29-1Jβ2-7 **(E)**, and risk score **(F)**.

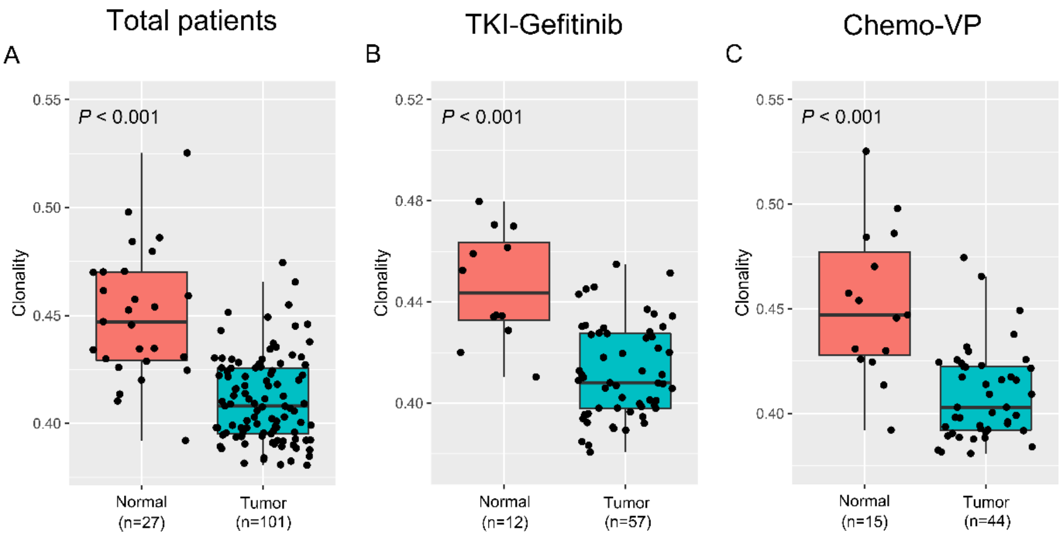

Figure 2. The level of clonality in the tumor-adjacent tissue and tumors in total patients **(A)**, and the TKI-Gefitinib **(B)** and Chemo-VP **(C)** cohorts. The line within the box indicates the median value, and lower and upper hinges represent 25% and 75% quantile, respectively.

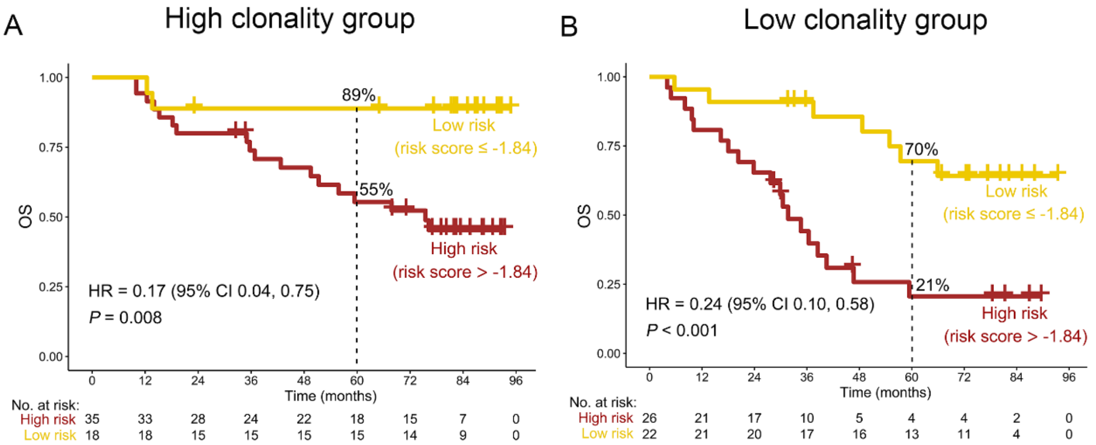

Figure 3. Kaplan-Meier curves for the high **(A)** and low **(B)** clonality groups were plotted based on low- and high-risk scores. HR, hazard ratio; CI, confidence interval.

Table 1. Clinical characteristics of NSCLC patients (n=101).

Factors	Cohorts		*P* value
	Gefitinib, N (%) [a]	VP, N (%) [a]	
Total	57	44	NA
Sex			0.925
Female	25 (43.9)	18 (40.9)	
Male	32 (56.1)	26 (59.1)	
Age (years) median (range)	59 (39 to 74)	61 (39 to 73)	0.359
Smoking history			0.605
Never	40 (70.2)	33 (75.0)	
Ever	9 (15.8)	4 (9.1)	
Current	8 (14.0)	7 (15.9)	
Pathology			0.255
Adenocarcinoma	53 (93.0)	44 (100%)	
Squamous cell carcinoma	3 (5.3)	0 (0)	
Other	1 (1.8)	0 (0)	
Clinical stage			0.111
II	16 (28.1)	19 (43.2)	
III	41 (71.9)	24 (54.5)	
Unknown	0 (0)	1 (2.3)	
N stage			0.119
N1	17 (29.8)	19 (43.2)	
N2	40 (70.2)	24 (54.5)	
Unknown	0 (0)	1 (2.3)	
Tumor-adjacent tissue	12	15	NA

a: Unless specifically mentioned; SD: Standard deviation; NA, not available.
